# Visual search for feature conjunctions: an fMRI study comparing alcohol-related neurodevelopmental disorder (ARND) to ADHD

**DOI:** 10.1186/s11689-015-9106-9

**Published:** 2015-03-04

**Authors:** Carrie R O’Conaill, Krisztina L Malisza, Joan L Buss, R Bruce Bolster, Christine Clancy, Patricia Dreessen de Gervai, Albert E Chudley, Sally Longstaffe

**Affiliations:** Department of Physiology, University of Manitoba, 432 Basic Medical Sciences Bldg, 745 Bannatyne Ave, Winnipeg, MB R3E 0J9 Canada; Department of Psychology, University of Manitoba, P404 Duff Roblin Bldg, 190 Dysart Rd, Winnipeg, MB R3T 2N2 Canada; National Research Council of Canada - Institute for Biodiagnostics, 435 Ellice Avenue, Winnipeg, MB R3T 1Y6 Canada; Department of Psychology, University of Winnipeg, 515 Portage Ave, Winnipeg, R3B 2E9 Canada; Division of Rehabilitation Psychology, Seattle Children’s Hospital, 4800 Sand Point Way, Seattle, WA 98105 USA; Department of Paediatrics, University of Manitoba, 336 Basic Medical Sciences Bldg, 745 Bannatyne Ave, Winnipeg, MB R3E OJ9 Canada; Department of Biochemistry and Medical Genetics, University of Manitoba, 336 Basic Medical Sciences Bldg, 745 Bannatyne Ave, Winnipeg, MB R3E OJ9 Canada; Department of Pediatrics and Child Health, CE-203 Children’s Hospital, Health Sciences Centre, 840 Sherbrook Street, Winnipeg, MB R3A 1S1 Canada

**Keywords:** Fetal alcohol spectrum disorder (FASD), Alcohol-related neurodevelopmental disorder (ARND), Attention-deficit/hyperactivity disorder (ADHD), Functional magnetic resonance imaging (fMRI), Attention, White matter, Gray matter, Diffusion tensor imaging (DTI), Inferior longitudinal fasciculus (ILF), Tract-based spatial statistics (TBSS)

## Abstract

**Background:**

Alcohol-related neurodevelopmental disorder (ARND) falls under the umbrella of fetal alcohol spectrum disorder (FASD). Diagnosis of ARND is difficult because individuals do not demonstrate the characteristic facial features associated with fetal alcohol syndrome (FAS). While attentional problems in ARND are similar to those found in attention-deficit/hyperactivity disorder (ADHD), the underlying impairment in attention pathways may be different.

**Methods:**

Functional magnetic resonance imaging (fMRI) and diffusion tensor imaging (DTI) was conducted at 3 T. Sixty-three children aged 10 to 14 years diagnosed with ARND, ADHD, and typically developing (TD) controls performed a single-feature and a feature-conjunction visual search task.

**Results:**

Dorsal and ventral attention pathways were activated during both attention tasks in all groups. Significantly greater activation was observed in ARND subjects during a single-feature search as compared to TD and ADHD groups, suggesting ARND subjects require greater neural recruitment to perform this simple task. ARND subjects appear unable to effectively use the very efficient automatic perceptual ‘pop-out’ mechanism employed by TD and ADHD groups during presentation of the disjunction array. By comparison, activation was lower in ARND compared to TD and ADHD subjects during the more difficult conjunction search task as compared to the single-feature search. Analysis of DTI data using tract-based spatial statistics (TBSS) showed areas of significantly lower fractional anisotropy (FA) and higher mean diffusivity (MD) in the right inferior longitudinal fasciculus (ILF) in ARND compared to TD subjects. Damage to the white matter of the ILF may compromise the ventral attention pathway and may require subjects to use the dorsal attention pathway, which is associated with effortful top-down processing, for tasks that should be automatic. Decreased functional activity in the right temporoparietal junction (TPJ) of ARND subjects may be due to a reduction in the white matter tract’s ability to efficiently convey information critical to performance of the attention tasks.

**Conclusions:**

Limited activation patterns in ARND suggest problems in information processing along the ventral frontoparietal attention pathway. Poor integrity of the ILF, which connects the functional components of the ventral attention network, in ARND subjects may contribute to the attention deficits characteristic of the disorder.

## Background

Fetal alcohol spectrum disorder (FASD), the umbrella term describing the spectrum of ethanol teratogenesis in humans, is a common cause of developmental disability [1]. At one end of this spectrum is fetal alcohol syndrome (FAS), characterized by growth deficiency, facial abnormalities, and central nervous system (CNS) damage. At the other end is a diagnosis of alcohol-related neurodevelopmental disorder (ARND). Individuals diagnosed with ARND frequently do not demonstrate many of the facial features characteristic of FAS, but still have neurodevelopmental and/or cognitive or behavioral abnormalities [1,2]. Executive functions, including working memory and response inhibition, and sustained attention are significantly affected by prenatal alcohol exposure [3-8]. CNS damage, manifested as anatomical, cognitive, and behavioral deficits, is diverse [9-11], and FASD diagnosis is a challenge, especially in individuals with ARND, as many of the symptoms are non-specific and no consistent neurodevelopmental profile has been established. Additionally, individuals diagnosed with ARND present with cognitive and behavioral deficits that overlap with other conditions, including attention-deficit/hyperactivity disorder (ADHD) [12-14]. Attentional problems found in FASD are similar to those found in individuals with ADHD [15,16]. Confounding this further is the likelihood that comorbid conditions exist within some individuals [12-14]. While the symptoms of ARND and ADHD are similar, the underlying impairments in cognitive pathways may be different.

The neurocognitive and behavioral control of attention is very complex. Posner divides attention into three subsystems: orienting, alerting, and detecting [17]. The current study focused on detection, which involves attending to signals used for focal or conscious processing [17]. Visual attentional processing occurs via two independent pathways in the extrastriate visual cortex, referred to as the dorsal and ventral streams [18]. The posterior parietal cortex, which is part of the dorsal visual stream, is involved in the attention to space and the location of objects. Lateral and inferior temporal regions belong to the ventral stream, which is responsible for feature-based processing and object recognition [18-20]. In turn, these systems are connected with dorsal and ventral portions of the dorsolateral frontal cortex, respectively [21,22]. The connection to the dorsolateral frontal cortex provides executive control over attentional processing and modulates the response of neurons in the dorsal and ventral stream so that attention is directed to places and objects within the environment that are relevant to the task at hand. Corbetta and Shulman further suggested that the task of selecting and attending to a target is shared between dorsal and ventral frontoparietal attention networks, working cooperatively. In this theory, the dorsal attention network is involved in top-down and goal-directed selection of stimuli. The ventral frontoparietal attention network is involved in the capture of attention by an unexpected or salient stimulus and represents a bottom-up or stimulus-driven attentional mechanism [23]. The cortex at the temporoparietal junction (TPJ) lies at the anatomical intersection between the dorsal and ventral streams. It is proposed that within the ventral frontoparietal network, the frontal cortex may be required to evaluate the salience of the stimuli and the TPJ may be involved in determining the behavioral importance of the stimuli [23].

This study was designed to elucidate differences between ARND and ADHD subjects in brain function related to attentional processes. We hypothesize that the attention problems in subjects prenatally exposed to alcohol result from different attentional mechanisms than those found in ADHD subjects. In the current study, we used functional magnetic resonance imaging (fMRI) to compare disjunction to conjunction search in individuals with ARND and ADHD, compared to typically developing (TD) subjects. We also used diffusion tensor imaging (DTI) to examine connections between cortical areas associated with the control of attention. This study was part of a larger experiment including fMRI of working memory [5], two attention tasks (the conjunction search task reported here and a spatial cueing task) and a response inhibition task and DTI.

## Methods

### Recruitment

Experimental protocols were reviewed and approved by the affiliated ethics boards (National Research Council - IBD Ethics Board, The University of Manitoba Ethics Board, and The University of Winnipeg ethics board).

A total of 63 subjects aged 10 to 14 years participated in the study (ARND (*n* = 22); ADHD (*n* = 20); TD (*n* = 21)). ARND subjects (*n* = 22) were recruited through the Manitoba FASD Centre (formerly the Clinic for Alcohol and Drug Exposed Children). Diagnosis of ARND includes extensive testing by a multidisciplinary team using established FASD diagnostic guidelines [1]. All 22 subjects completed the conjunction task. Of these, one was excluded from the DTI portion of the study due to image artifact problems and one subject did not complete the DTI portion study (*n* = 20 DTI).

Subjects with ADHD (*n* = 20) were diagnosed and referred through pediatricians. These diagnoses involved clinical assessments including history, physical examination, and review of home and school behaviors/academic achievement with diagnosis based on criteria in the Diagnostic and Statistical Manual (DSM)-IV. One of these subjects did not complete the conjunction task sufficiently well to be included in the analysis (*n* = 19 conjunction) but did complete the DTI study (*n* = 20).

Typical control subjects (*n* = 21) were recruited through public posters and word of mouth. All of the TD subjects completed both the conjunction and DTI portion of the study. Additionally, all subjects in the ADHD and TD groups were evaluated using parental questionnaires to ensure they were not exposed prenatally to alcohol. TD volunteers were matched with ADHD and ARND subjects on the basis of age, sex, intelligence quotient (IQ), and socioeconomic status (SES; average household income in the postal code of residence). The demographics for the participants who completed the fMRI conjunction task are presented in Table 1. All subjects were free of stimulant medication (methylphenidate, dextroamphetamine, and caffeine) for at least 36 h prior to the fMRI acquisition and for the psychological assessments. Subjects who were prescribed Strattera, risperidone, or other non-stimulant medication continued to take these medications for the study.

**Table 1:** **Subject characteristics for the conjunction task**

**Measure**	**TD (** ***n*** **= 21)**		**ARND (** ***n*** **= 22)**		**ADHD (** ***n*** **= 19)**		**Significance** ^**a**^	***Post hoc*** **tests**
	**Mean**	**Std dev**	**Mean**	**Std dev**	**Mean**	**Std dev**		
Age (years)	12.60	1.29	12.20	1.64	11.93	1.33	*F* = 1.098, *p* = 0.340	
Sex (% male)	76.2		63.6		89.5		*χ* ^*2*^ = 3.713, *p* = 0.156	
Household income ($)	70,842	15,302	49,733	21,118	65,287	27,879	*F* = 5.261, *p* = 0.008	TD > ARND, *p* = 0.007^b^
Full-scale IQ	107.81	13.08	73.05	12.33	95.79	16.97	*F* = 32.527, *p* < 0.001	TD > ARND, *p* < 0.001^c^
								ADHD > ARND, *p* < 0.001^c^

^a^ *p* value cutoff = 0.05; ^b^Games-Howell test; ^c^Tukey’s HSD test.

Of the subjects in the ARND group who completed the fMRI tasks, five were not prescribed any medication at the time of the study, four were taking Concerta, and four were taking Dexedrine and were off these medications for the study. In addition, four subjects were taking both Concerta and risperidone (one subject off both medications for the study); one subject was taking Strattera; one Seroquel; one Concerta (off for study), risperidone, and olanzapine; one was taking Dexedrine (off for study), Seroquel, risperidone, and Prozac; and one was taking Adderall (off for study), fluoxetine, and risperidone.

Four children in the ADHD who completed the fMRI tasks were not prescribed any medications. Nine children were prescribed either Concerta or Ritalin alone or in combination and one subject on Dexedrine; these subjects were off these medications for the study. Three subjects were taking Concerta and/or Ritalin (off for study) and risperidone (one off all medications for the study) and one was taking risperidone and Strattera. One child was prescribed asthma medication.

### Comorbidities

Comorbid disorders are often associated with FASD [14] with the most common comorbidity being ADHD [12,13]. Nine subjects in the ARND group were diagnosed with ADHD, while the remaining ARND subjects had no documented comorbid disorders. Typically, ADHD within FASD is more likely to be the earlier-onset, inattentive subtype, with comorbid developmental psychiatric and medical conditions such as anxiety, mood, conduct, or explosive disorders [12]. Several of the ARND subjects diagnosed with ADHD exhibited other comorbid diagnoses including one subject with learning disability, conduct disorder, and global delay and one subject with oppositional defiant disorder (ODD), depression, and attachment disorder. The ARND/ADHD comorbidity is controversial since attentional deficits are relevant for an ARND diagnosis [1]. However, due to the frequency of ARND/ADHD comorbid diagnoses, it is inadvisable to exclude these subjects due to the sample bias that would result. The definition of ARND and ADHD (ARND/ADHD^+^) in the present study means that the subjects met the diagnostic criteria for both ARND (through the multidisciplinary diagnostic approach used) and ADHD (as defined above, including the criteria in the DSM-IV) during their clinical evaluation. The ADHD group included four subjects with learning disabilities (math and/or reading) and one subject with ODD, which are typical of the many learning and behavioral disorders associated with ADHD. None of the subjects in the TD group were diagnosed with any learning or behavioral disorders, and none had confirmed prenatal alcohol exposure.

### Psychological assessment

All subjects were administered the *Wechsler Intelligence Scale for Children-Fourth Edition* (*WISC-IV*; 2003, Pearson Canada, Toronto, ON) and the *Conners’ Continuous Performance Test II* (*CPT-II*; MHS Inc, Toronto, ON). Teachers and parents/caregivers completed several standardized behavior rating forms, including the *Conners’ Rating Scales*, the *Child Symptom Inventory-Fourth Edition* (*CSI-4*) or the *Adolescent Symptom Inventory-Fourth Edition* (*ASI-4*) (Pearson Canada, Toronto, ON), and the *Behavior Rating Inventory of Executive Function* (*BRIEF*; WPS, Torrance, CA).

### fMRI tasks

Triesman and colleagues’ feature integration theory proposed two levels of visual processing: 1) pre-attentive processing, which is automatic and processes visual features in parallel, and 2) focused attention processing, which is consciously controlled and involves serial search [24-26]. In the current experiment, we used a visual search task modeled after Treisman’s conjunction search paradigm. Triesman and colleagues have shown that in the disjunction condition, when the distractor differs from the target in both color and letter, the target would immediately ‘pop-out’ and did so independently of the number of distractor items present. They argued this to be a bottom-up process, where all stimuli were searched in parallel within a single ‘feature map’ [26]. However, when participants searched for the target in a task containing distractors that possessed a combination of features (i.e., the target differed from the distractors in color and orientation), the participants had to switch between feature maps in a controlled or top-down fashion. Triesman argued that conjunction search thus engaged a serial search process, wherein each location had to be searched on a separate feature map and search time increased linearly as a function of the number of items in the array [26]. This serial process requires more effort or allocation of attentional resources compared to the bottom-up, parallel processing associated with pop-out in feature disjunction [27].

**Figure 1:**
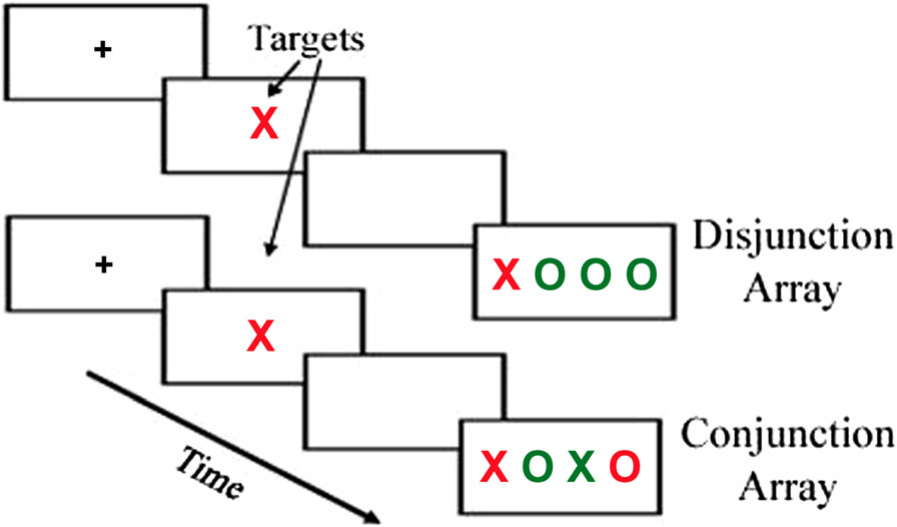
**Illustration of the fMRI task depicting the conjunction and disjunction conditions.**

An event-related fMRI feature-based visual search task was completed by each subject to assess the attention systems. Compound trials began with the presentation of a fixation cross in the middle of the screen for 500 ms. Next, a colored letter target was presented in the middle of the screen for 500 ms; this target was either a red- or green-colored X or O and was followed by a blank screen presented for 1,000 ms. Finally, a horizontal array consisting of four colored letters (one of which was the target) was presented for 500 ms. Subjects indicated the position of the target within the array by pressing a button with their right hand that corresponded to its location. The number of features shared between distractors and the target were varied. In the disjunction array, the target was both a different color and a different letter than the distractors (i.e., distinct in both features). By comparison, in the conjunction array, the target shared one common feature with two of the distractors in the array and was unique only in the combination of both target features. For example, if the target was a red ‘X’, the array consisted of the target (a red ‘X’), a green ‘X’, a red ‘O’, and a green ‘O’. Figure 1 illustrates the fMRI task, depicting the conjunction and disjunction arrays.

The paradigm consisted of 64 trials. Both array type and target features were varied pseudorandomly across trials and counterbalanced for frequency of presentation. Trials were separated by a jittered interstimulus interval (ISI) of 2 to 8 s. Response times were calculated from the onset of the horizontal array. Subjects were instructed to respond as quickly and accurately as possible for all trials. Reaction times and accuracy were recorded for each array type. The paradigm was practiced prior to imaging until subjects understood and could perform the tasks accurately.

### Statistics for psychological tests and task performance

Statistics were calculated using Statistical Package for Social Sciences (SPSS 19.0, IBM, Armonk, NY). In many cases, task performance data could not be transformed to meet the assumptions of parametric statistics. Instead of repeated-measures analyses of variance (ANOVAs), one-way ANOVAs followed by Games-Howell *post hoc* tests were calculated, as well as paired *t*-tests as necessary. Instead of ANCOVAs, *Z* scores were calculated. In all cases, *p* values were corrected for multiple comparisons.

### Magnetic resonance imaging

All children were trained in a mock scanner prior to the MRI session to ensure both their comfort and their ability to remain motionless.

All imaging experiments were conducted on a 3 T Siemens TIM Trio MRI system (Siemens, Erlangen, Germany). Functional MRI data were acquired using a single shot, gradient echo-echo planar imaging (EPI) sequence with a matrix size of 64 × 64, field of view (FOV) of 240 mm, echo time (TE) of 40 ms, and repetition time (TR) of 2 s. With motion correction on, 28 interleaved 5-mm-thick contiguous slices were acquired along the anterior commissure-posterior commissure (AC-PC) line, resulting in full brain coverage and a voxel size of 3.75 × 3.75 × 5 mm. A total of 243 volumes were acquired. Standard T1-weighted images were acquired with the same slices selected as for the fMRI experiments. Functional MRI data were analyzed using SPM5 (http://www.fil.ion.ucl.ac.uk/spm/). Data were converted to SPM analyze format, reoriented, realigned, normalized to the Montreal Neurological Institute (MNI) EPI template, and smoothed with a 6-mm isotropic kernel. Activation was assessed using an event-related model through the change in signal intensity and volume of activated clusters following the modeled time course (canonical hemodynamic response function (HRF) synchronized to the onset of the array) over the whole brain.

Data were analyzed using fMRI scans corresponding to trials in which the subjects responded correctly. For one ADHD subject, a sequence of scans corresponding to a period in which the subject was not engaged in the task was manually removed from the analysis. Within-group analysis was conducted using one-sample *t*-tests using the individual subjects’ images for the contrasts of interest. Pair-wise between-group comparisons were completed using ANOVAs (*p* < 0.0167). Significance levels were corrected for multiple comparisons using cluster correction yielding family-wise errors (FWE) as indicated for each comparison [28].

### DTI

In order to obtain the required DTI information for all tasks in the overall study (attention, working memory, and response inhibition pathways), it was necessary to acquire images across the full brain. Due to the nature of the affected populations, it was also necessary to acquire high-quality DTI within a short period of time (less than 5 min) to avoid motion artifacts that would compromise study results. Therefore, DTI was performed using a spin echo-echo-planar imaging (SE-EPI) sequence, and the following parameters were used: 240 FOV, 128 × 128 matrix size, 27 5-mm-thick slices (resulting in a 1.89 × 1.89 × 5 mm voxel size) acquired interleaved on an axial plane with anterior-posterior phase encoding, four averages, TR = 3,700 ms, TE = 93 ms, and bandwidth of 1,396 Hz/Px. Two diffusion weightings (*b* = 0 and 1,000 s/mm^2^) and 20 gradient directions were used. A 3D magnetization prepared rapid gradient echo (MPRAGE) anatomical image was acquired (256 × 256 matrix, 256 mm FOV, inversion time (TI) = 900 ms, TR = 1,900 ms, TE = 2.2 ms, bandwidth = 2,332 Hz/Px). Following eddy current correction and brain extraction, the diffusion tensors were calculated using DTIFIT (FSL; FMRIB Software, University of Oxford, Oxford, UK). Voxel-wise statistical analysis of fractional anisotropy (FA) and mean diffusivity (MD) data were then completed using tract-based spatial statistics (TBSS) [29]. Areas of white matter showing significant FA and MD differences (*p* < 0.05) between the three groups were determined using pair-wise comparisons, allowing an examination of white matter integrity.

## Results

### Demographics and behavioral data

The TD (*n* = 21), ARND (*n* = 22), and ADHD (*n* = 19) groups were well-matched for age and sex distribution (Table 1). Full-scale IQ (FSIQ) was significantly lower in the ARND group than both the TD and ADHD groups, a finding typical of FASD subjects [30]. SES, represented by the average household income in the neighborhood of residence, was significantly lower in the ARND group than the TD group.

Inattention scales were obtained for each subject using the CPT-II (errors of omission (OM), cognitive/inattention) and DSM-IV. ARND subjects had higher OM scores than the TD and ADHD groups, and both clinical groups had significantly higher scores than controls on the two measures of inattention (Table 2). Across all subjects, conjunction search accuracy was significantly correlated with OM (Spearman’s *ρ* = −0.591, *p* < 0.001), cognitive/inattention (Spearman’s *ρ* = −0.529, *p* < 0.001), and DSM-IV inattention (Spearman’s *ρ* = −0.599, *p* < 0.001) scores, consistent with the assumption that conjunction search involves attention and working memory [24,25,27].

**Table 2:** **Psychological tests and fMRI task performances**

**Parameter**	**TD (** ***n*** **= 21)**		**ARND (** ***n*** **= 22)**		**ADHD (** ***n*** **= 19)**		**Significance** ^**a**^	***Post hoc*** **tests** ^**b**^
	**Mean**	**Std dev**	**Mean**	**Std dev**	**Mean**	**Std dev**		
Cognitive/inattention scores	49.0	5.2	71.9	11.6	65.7	11.3	*F* = 30.166, *p* < 0.001	TD < ARND, *p* < 0.001
								TD < ADHD, *p* < 0.001
DSM-IV inattention scores	49.4	5.2	71.7	12.4	65.8	10.9	*F* = 27.809, *p* < 0.001	TD < ARND, *p* < 0.001
								TD < ADHD, *p* < 0.001
Errors of omission (OM)	45.4	5.2	59.3	13.9	48.3	7.4	*F* = 11.723, *p* < 0.001	ARND > TD, *p* = 0.001
								ARND > ADHD, *p* = 0.012
Disjunction accuracy (%)	94.6	6.0	86.0	11.4	93.4	5.4	*F* = 6.876, *p* = 0.002	ARND < TD, *p* = 0.011
								ARND < ADHD, *p* = 0.030
Conjunction accuracy (%)	91.7	7.6	71.0	13.9	84.3	10.9	*F* = 18.867, *p* < 0.001	ARND < TD, *p* < 0.001
								ARND < ADHD, *p* = 0.004
Conjunction errors (%)	5.2	6.8	18.3	10.6	9.2	8.9	*F* = 12.083, *p* < 0.001	ARND > TD, *p* < 0.001
								ARND > ADHD, *p* = 0.013
Conjunction non-response (%)	3.1	3.0	10.9	11.3	8.0	8.9	*F* = 4.597, *p* = 0.014	
Disjunction response time (ms)	729.3	184.2	819.9	184.0	804.0	161.3	*F* = 1.569, *p* = 0.217	
Conjunction response time (ms)	799.0	178.1	883.0	186.0	854.5	187.3	*F* = 1.153, *p* = 0.323	
Disjunction response variability (ms)	135.8	77.9	220.3	116.0	250.0	130.7	*F* = 5.933, *p* = 0.004	ARND < ADHD, *p* = 0.021
								ADHD > TD, *p* = 0.007
Conjunction response variability (ms)	172.0	67.1	248.1	87.3	251.5	144.8	*F* = 3.943, *p* = 0.025	

^a^Bonferroni-corrected *p* value for multiple comparisons = 0.005; ^b^Games-Howell test.

### fMRI task performance

It was critical to use tests that were simple and allowed children in the affected groups to perform them with relative ease. Accuracy for visual search of the conjunction and disjunction arrays was high in all three groups (Table 2). ARND subjects performed significantly worse than the ADHD and TD groups for both search tasks. Although differences in accuracy were statistically significant, the number of errors and thus the number of trials excluded from the fMRI analysis was not high enough to introduce significant bias into the analysis. Accuracy for the conjunction and disjunction searches were equal for TD subjects (paired *t* = −1.943, *p* = 0.066), whereas accuracy was lower for the challenging conjunction task in both ARND (paired *t* = −3.414, *p* = 0.003) and ADHD (paired *t* = −6.969, *p* < 0.001) subject groups.

Although differences in performance were noted between groups, they were minimized by using an event-related design allowing analysis of correct responses only. This facilitated comparison of cognitive processes across all three groups and ensured that all fMRI activation patterns were directly related to the task at hand and reliably showed attentional processing during each feature search task. Accuracies were above 70% for all subjects on the fMRI task, including the more difficult conjunction visual search.

It was expected that reaction time would be faster for disjunction search because of its more automatic pop-out characteristics compared to the top-down processing requirements of the more challenging conjunction search [24,26]. This relationship was observed in the TD and ARND subjects (TD: paired *t* = 6.493, *p* < 0.001; ARND: paired *t* = 4.476, *p* < 0.001), but was not seen in the ADHD group (ADHD: paired *t* = 2.297, *p* = 0.034). However, the variability of response time (standard deviation of the response time, RTSD) of disjunction search was significantly higher in the ADHD group compared to the TD and ARND groups (Table 2).

### fMRI results

#### Disjunction search

**Figure 2:**
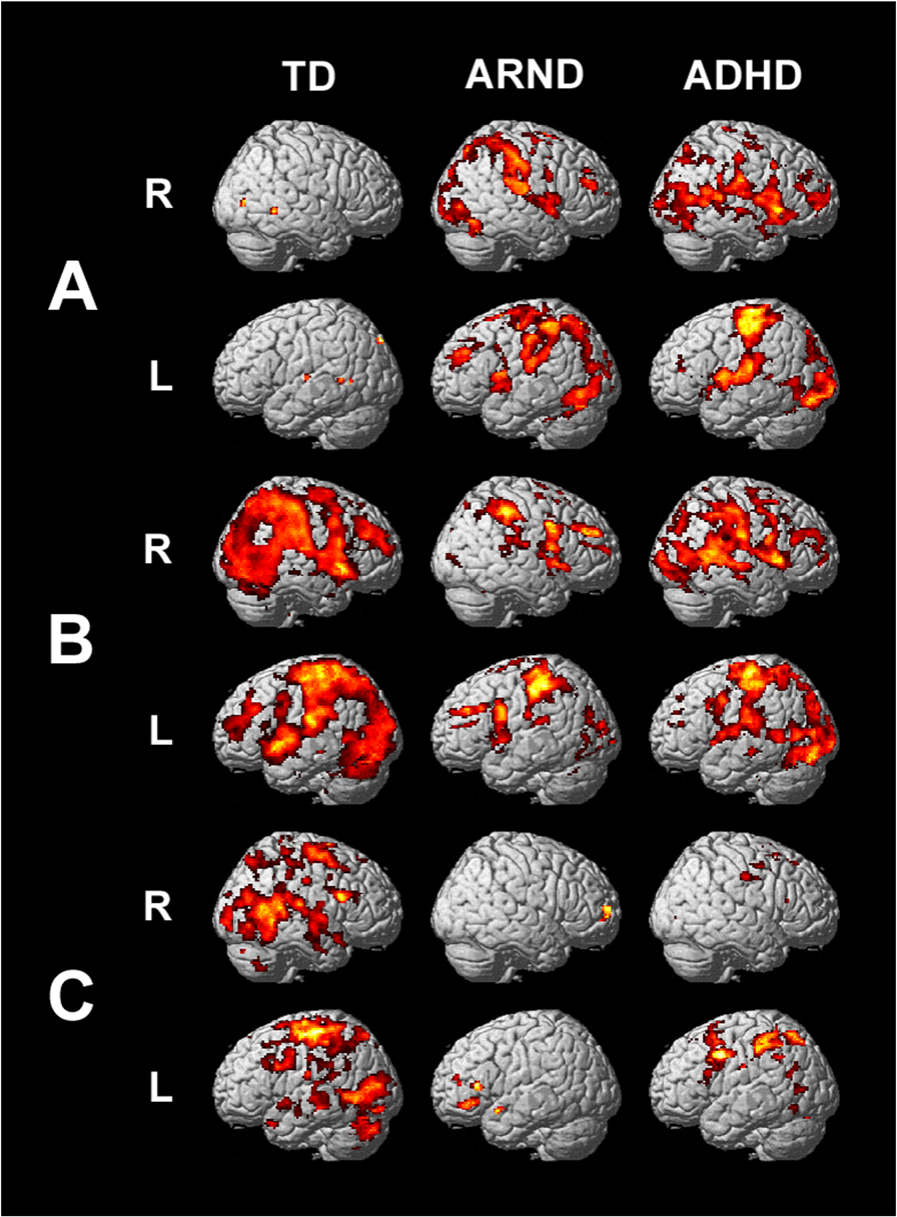
**fMRI activation maps.** fMRI activation maps for **(A)** the disjunction contrast (FWE *p* = 0.001, corrected for multiple comparisons using cluster size = 10), **(B)** the conjunction contrast (FWE *p* = 0.001, corrected, cluster size = 10), and **(C)** the conjunction minus disjunction contrast (FWE *p* = 0.05, corrected, cluster size = 22). R, right; L, left. The color of the activations corresponds to increased significance with increasingly hot colors (black-red-yellow-white); activations range from *T* = 1.9 (*p* = 0.05) for **(C)** and *T* = 3.9 (*p* = 0.001) for **(A)** and **(B)** to a maximum of *T* = 14.

Figure 2A shows the activations for the three groups observed during the disjunction search task. Only very small fMRI activations were observed for the TD group during the disjunction visual search task. These activations were limited to the right posterior cingulate gyrus (Brodmann areas (BA) 23 and 31), bilateral thalami, bilateral middle temporal gyrus (BA 21), left superior temporal gyrus (BA 22), left precuneus (BA 7), middle frontal (BA 10), and right middle occipital gyrus (BA 19) (Figure 2A, corrected *p* < 0.001). It should be noted, however, that there were extensive activations of the whole ventral and dorsal attention pathways at lower, but still significant, thresholds (not shown). The fMRI task performance measures and the behavioral measures from the psychological testing are found in Table 2. Both the fMRI and behavioral measures support the assumption that visual search of the disjunction array is very efficient due to automatic perceptual pop-out (Table 2) [24,26] (i.e., the disjunction search resulted in little cortical activation in TD subjects). Compared to TD subjects, subjects diagnosed with ARND had much more extensive fMRI activations along the dorsal frontoparietal network during the disjunction search, including areas consistent with visuospatial attention and motor responses (Figure 2A, corrected *p* < 0.001).

The overall pattern of activations for the ADHD group during the disjunction search task was similar to that of the ARND group (Figure 2A). Cortical activations were found along both the dorsal and ventral attention pathways in ADHD subjects, with the TPJ being activated bilaterally. Activations in the left superior frontal gyrus (BA 6 and 8), medial frontal gyrus (BA 46 and 10), and cingulate (BA 23, 24 and 31) were stronger for subjects diagnosed with ARND than either ADHD or TD subjects (data not shown). The ADHD group had stronger activations in the right inferior frontal gyrus (BA 45 and 47), right middle temporal gyrus (BA 39 and 37), and left occipital regions (BA 18 and 19) compared to TD and ARND groups (data not shown).

#### Conjunction search

Figure 2B shows activations in the three subject groups during the conjunction search task. In TD subjects, an overall increase in cortical activations throughout the ventral and dorsal frontoparietal attention networks was observed during the conjunction search (Figure 2B, corrected *p* < 0.001). The substantial activations for the conjunction task extended through the frontal, parietal, temporal, and lateral occipital cortices. Figure 2C shows the subtraction contrast (conjunction minus disjunction) for the three subject groups. Subtracting the activation related to disjunction search removed common activations in the occipital and superior parietal regions, leaving activation concentration in the TPJ, superior paracentral region, and areas of the inferior frontal and superior temporal lobe (Figure 2C). Specific regions of increased activation in the subtractive contrast of the TD group were observed across the bilateral precentral gyrus (BA 4 and 6), right post-central gyrus (BA 2, 3, and 40), left superior temporal gyrus (BA 38 and 22), bilateral middle temporal (BA 21), bilateral inferior frontal (BA 47 and right BA 45), right superior frontal gyrus (BA 8), bilateral cuneus (BA 18 and 19), right insula (BA 13), and right parahippocampal gyrus (BA 34) (Figure 2C). These activations correspond to important areas within both the dorsal and ventral frontoparietal attention network. This increased amount of functional activation during the conjunction search task is consistent with the increased attentional recruitment required to complete the task [24-26].

In ARND subjects, the conjunction search elicited similar activations as the disjunction search (Figure 2B). Consequently, the subtractive contrast (Figure 2C) revealed minimal activation, with task-related increases confined primarily to the left prefrontal cortex (BA 45, 46, and 47), left superior temporal gyrus (BA 38), right cuneus (BA 18), bilateral middle and right superior frontal gyrus (BA 10), and bilateral caudate. Cortical activation in the ARND subject group was significantly lower than that in the TD group in areas of the bilateral precentral gyri (BA 6), bilateral parietal including post-central gyrus (BA 5 and 7), left middle frontal (BA 6, 8, and 9) and right middle and superior frontal areas (BA 8), left superior temporal (BA 22), left insula (BA 13), and anterior and posterior cingulate (BA 30, 31, and 33).

Analysis of the subtractive contrast in the ADHD subject group (Figure 2C) revealed that conjunction search generated slightly stronger activations than disjunction search in the left ventrolateral prefrontal cortex (BA 45), bilateral superior frontal, middle frontal and precentral gyri (BA 6, 8, and 9), bilateral post-central parietal region (BA 2 and 3), anterior and posterior cingulate (BA 24, 30, and 33), bilateral precuneus (BA 7), bilateral occipital regions (BA 18 and 19), left inferior parietal (BA 40), right insula (BA 13), left fusiform gyrus (BA 37), and left superior and middle temporal regions (BA 39).

**Figure 3:**
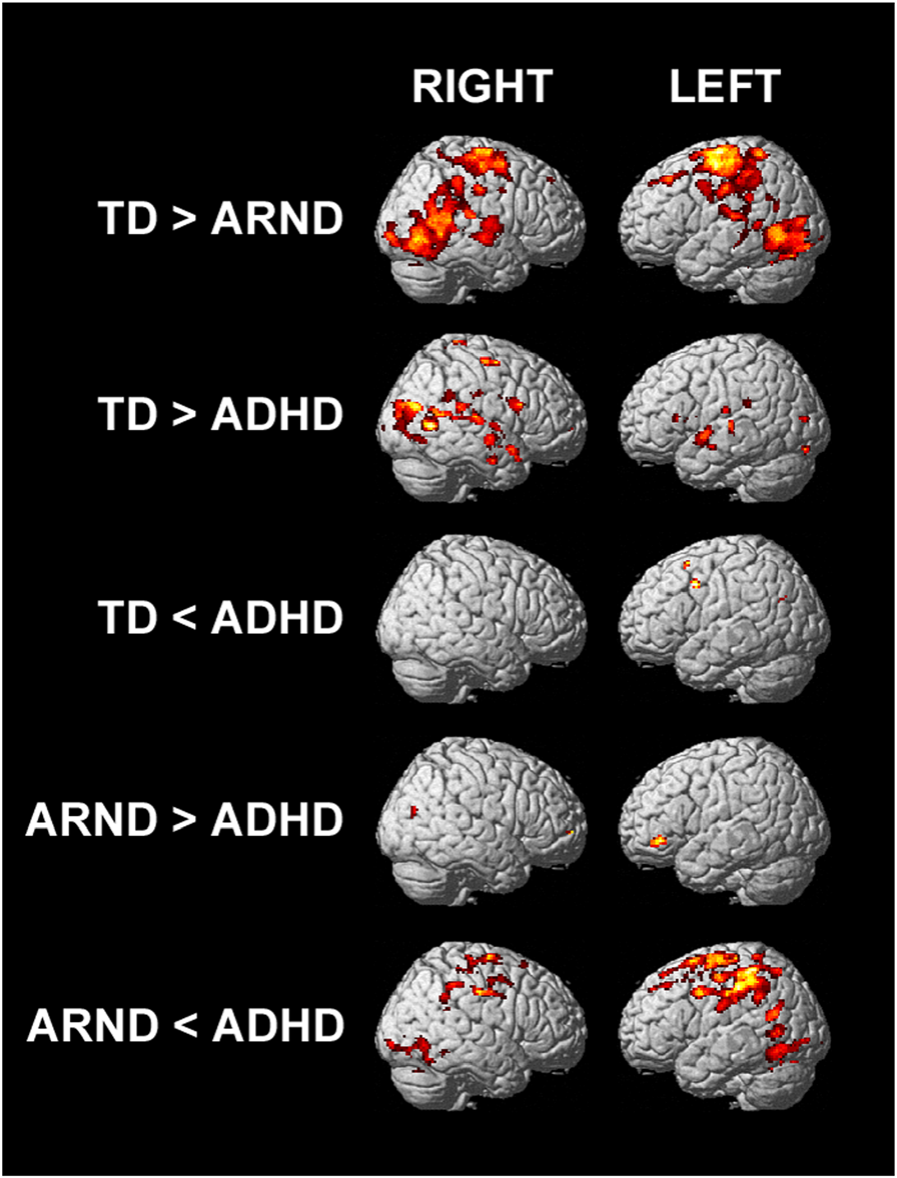
**fMRI activation maps for the pair-wise comparisons of the conjunction-disjunction contrast.** FWE *p* = 0.0167, corrected for multiple comparisons using cluster size = 12. The color of the activations corresponds to increased significance with increasingly hot colors (black-red-yellow-white); activations range from *T* = 1.9 to 4.4.

Figure 3 shows the fMRI activation maps for the pair-wise (ANOVA) group comparisons of the conjunction minus disjunction contrast. Activations in the ADHD group were larger than in the ARND group following subtraction of the disjunction task from the conjunction task, but were more restricted in area as compared to the TD group (Figure 2C; Tables 3, 4, 5, and 6). Several areas of the anterior and superior temporal lobe that were activated in the TD group were notably absent in the ADHD group. An area of activation persisted in the TPJ in the ADHD group during the subtractive contrast; however, the level of activation was reduced in comparison to that observed in the TD group.

**Table 3:** **Regions of greater activation for TD subjects than ADHD subjects after conjunction-disjunction subtractive analysis**

**Region**	**Hemisphere**	**Gyrus**	**BA**	**Talairach coordinates**			**Voxel** ***T***	**Cluster extent**
				***X***	***Y***	***Z***		
Frontal	Left	Inferior frontal	13	−36	22	8	3.27	184
		Inferior frontal	47	−26	7	−17	2.56	14
		Paracentral lobule	5	0	−39	66	2.64	33
		Precentral	44	−42	4	9	2.37	12
	Right	Inferior frontal	45	55	15	20	3.03	103
		Medial frontal	6	8	−28	70	2.78	17
		Middle frontal	6	34	−3	54	2.72	121
		Paracentral lobule	6	2	−30	57	2.59	25
		Precentral	6	38	−11	56	3.36	121
		Precentral	6	42	−3	55	2.45	121
		Precentral	6	40	1	26	3.01	52
		Superior frontal	10	20	54	−1	2.64	14
Limbic	Left	Cingulate	31	−2	−36	26	2.98	114
	Right	Cingulate	24	4	0	41	2.59	18
	Right	Cingulate	31	32	−43	26	3.20	24
		Parahippocampal	19	40	−41	−3	2.78	22
		Parahippocampal	19	26	−45	−3	2.52	15
Temporal	Left	Fusiform	20	−38	−11	−23	2.50	13
		Sub-gyral	21	−44	−10	−11	2.86	270
		Superior temporal	22	−57	2	−5	4.10	270
		Superior temporal	22	−53	−23	3	2.53	57
	Right	Inferior temporal	20	48	−9	−21	2.82	101
		Middle temporal	21	57	−60	5	4.06	206
		Middle temporal	21	61	−10	−6	4.04	69
		Middle temporal	21	51	7	−15	2.79	91
		Middle temporal	21	38	−1	−23	2.47	101
		Middle temporal	21	48	−7	−15	2.45	101
		Middle temporal	39	44	−56	6	2.73	206
		Middle temporal	39	44	−71	24	3.89	451
		Middle temporal	39	46	−75	15	3.25	451
		Middle temporal	39	42	−52	15	3.27	568
		Sub-gyral	21	40	−3	−12	2.89	198
		Superior temporal	22	59	−56	14	2.71	206
		Superior temporal	22	51	−6	4	2.80	45
		Superior temporal	22	59	2	−3	2.33	45
		Superior temporal	38	42	12	−22	3.20	91
		Superior temporal	41	57	−17	8	3.09	568
Parietal	Left	Inferior parietal lobule	40	−63	−36	24	2.93	20
		Postcentral	40	−57	−19	18	2.65	23
		Precuneus	7	0	−49	39	3.36	56
	Right	Postcentral	3	20	−33	72	3.39	21
		Postcentral	5	6	−45	67	2.78	33
		Precuneus	31	4	−53	30	2.66	23
		Supramarginal	40	63	−41	30	2.92	36
Occipital	Left	Lingual	18	−12	−54	3	2.53	15
		Middle occipital	18	−20	−86	−9	2.81	30
		Middle occipital	19	−36	−83	13	2.83	25
	Right	Cuneus	18	12	−99	7	2.67	12
		Cuneus	30	10	−62	7	2.51	34
		Middle occipital	19	53	−64	−4	3.27	451
Sub-lobar	Left	Caudate: caudate body		−16	−14	21	3.97	101
		Caudate: caudate body		−6	20	6	3.38	389
		Caudate: caudate tail		−20	−40	15	3.33	49
		Claustrum		−38	−11	4	3.30	270
		Claustrum		−30	−5	17	2.55	22
		Insula	13	−34	−13	17	2.48	22
		Lentiform nucleus: medial globus pallidus		−14	−4	0	3.01	115
		Thalamus		−18	−7	17	2.62	101
		Thalamus		−6	−12	1	2.20	115
		Thalamus: pulvinar		−6	−29	12	3.10	103
	Right	Claustrum		36	−10	−8	3.03	198
		Insula	13	51	−34	20	3.11	568
		Insula	13	40	−9	6	2.78	198
		Lentiform nucleus: lateral globus pallidus		24	−15	6	2.82	84
		Lentiform nucleus: putamen		20	−7	15	2.87	389
		Thalamus		8	−10	2	2.79	84
		Thalamus		18	−14	1	2.50	84
		Thalamus: pulvinar		8	−27	11	3.01	103
		Thalamus: pulvinar		16	−34	13	2.51	103
		Thalamus: ventral lateral nucleus		18	−15	19	3.49	389
Midbrain	Left	Substantia nigra		−10	−10	−10	2.95	115
Anterior cerebellum	Left	Cerebellar lingual		−8	−43	−10	2.88	31
		Culmen		−18	26	−17	2.90	14

FWE *p* = 0.0167, corrected for multiple comparisons using cluster size = 12.

**Table 4:** **Regions of greater activation for ADHD subjects than TD subjects after conjunction-disjunction subtractive analysis**

**Region**	**Hemisphere**	**Gyrus**	**BA**	**Talairach coordinates**			**Voxel** ***T***	**Cluster extent**
				***X***	***Y***	***Z***		
Frontal	Left	Middle frontal	9	−42	8	36	2.96	32
		Precentral	9	−32	6	33	3.21	28
		Superior frontal	8	−38	16	53	2.79	16
Limbic	Right	Parahippocampal		28	−22	−12	3.06	15
		Hippocampus						
Temporal	Left	Middle temporal	39	−34	−59	27	2.51	21
Parietal	Left	Precuneus	39	−36	−66	31	2.23	21
Anterior cerebellum	Right			4	−42	−26	3.08	27

FWE *p* = 0.0167, corrected for multiple comparisons using cluster size = 12.

**Table 5:** **Regions of greater activation for ARND than ADHD subjects after conjunction-disjunction subtractive analysis**

**Region**	**Hemisphere**	**Gyrus**	**BA**	**Talairach coordinates**			**Voxel** ***T***	**Cluster extent**
				***X***	***Y***	***Z***		
Frontal	Left	Middle frontal	47	−44	37	−7	2.96	75
	Right	Superior frontal	10	20	54	−1	3.35	53
Temporal	Right	Middle temporal	39	48	−73	22	2.56	13

FWE *p* = 0.0167, corrected for multiple comparisons using cluster size = 12.

**Table 6:** **Regions of greater activation for ADHD than ARND subjects after conjunction-disjunction subtractive analysis**

**Region**	**Hemisphere**	**Gyrus**	**BA**	**Talairach coordinates**			**Voxel** ***T***	**Cluster extent**
				***X***	***Y***	***Z***		
Frontal	Left	Middle frontal	8	−36	33	41	3.27	69
		Middle frontal	9	−32	45	36	3.13	69
		Middle frontal	8	−44	20	45	2.38	13
	Right	Middle frontal	8	53	10	42	3.09	239
		Middle frontal	6	34	2	44	2.42	22
		Middle frontal	6	22	4	44	2.40	12
		Precentral	6	61	−16	38	2.91	239
		Superior frontal	6	14	20	58	2.42	17
Limbic	Left	Anterior cingulate	33	−8	20	17	2.60	12
		Cingulate	24	−12	4	44	3.68	7,529
		Posterior cingulate	30	−22	−60	5	3.61	4,219
	Right	Cingulate	24	16	−14	39	3.62	7,529
		Parahippocampal	35	18	−28	−9	3.17	148
Temporal	Left	Middle temporal	39	−50	−63	29	2.59	184
		Middle temporal	21	−57	−56	3	2.48	29
		Sub-gyral: hippocampus		−30	−27	−2	2.57	19
		Superior temporal	39	−50	−57	23	2.87	184
	Right	Sub-gyral: hippocampus		30	−24	−11	3.79	148
		Sub-gyral	37	46	−45	−10	3.20	61
		Hippocampus: dentate		20	−52	−23	2.60	50
Parietal	Left	Postcentral	2	−40	−22	34	3.88	7,529
	Right	Postcentral	3	48	−18	38	3.16	239
		Postcentral	2	36	−28	33	2.82	24
Occipital	Right	Cuneus	18	26	−93	1	2.81	45
		Lingual	18	8	−74	2	2.68	29
		Precuneus	31	24	−71	16	2.63	21
Sub-lobar	Right	Thalamus: pulvinar		22	−28	14	2.65	31
Posterior cerebellum	Left	Declive		−14	−76	−13	3.32	4,219
	Right	Declive		28	−69	−18	3.07	36
		Declive		36	−65	−20	2.80	36
		Declive		6	−67	−12	2.73	37
Anterior cerebellum	Left	Culmen		−30	−44	−20	3.59	4,219
		Culmen of vermis		−2	−62	−5	2.77	15
	Right	Culmen		14	−32	−22	2.72	40

FWE *p* = 0.0167, corrected for multiple comparisons using cluster size = 12.

It is notable that while ADHD subjects had greater precentral activations compared to ARND subjects (Table 6), TD subjects had more than either clinical group (Figure 2, Tables 3, 4 and 7). Like TD subjects, ADHD subjects had significantly greater activations in posterior regions of the ventral frontoparietal network (BA 6, 33, and 39) and occipital regions (BA 18) than ARND subjects (Tables 6 and 7).

**Table 7:** **Regions of greater activation for TD subjects than ARND subjects after conjunction-disjunction subtractive analysis**

**Region**	**Hemisphere**	**Gyrus**	**BA**	**Talairach coordinates**			**Voxel** ***T***	**Cluster extent**
				***X***	***Y***	***Z***		
Frontal	Left	Middle frontal	6	−26	−7	57	4.39	32,410
		Middle frontal	6	−51	2	39	3.46	152
		Middle frontal	8	−36	33	41	3.76	186
		Middle frontal	8	−30	22	45	2.68	186
		Middle frontal	9	−28	46	36	2.81	186
		Precentral	6	−46	−4	28	2.58	152
		Precentral	6	−34	3	22	2.97	60
	Right	Middle frontal	8	53	10	42	3.12	42
		Precentral	6	46	−2	46	2.52	42
		Precentral	6	57	4	33	2.74	19
		Precentral	6	42	−8	34	2.43	12
		Superior frontal	8	30	45	38	2.55	18
Limbic	Left	Cingulate	31	−14	−37	37	4.21	32,410
	Right	Anterior cingulate	33	4	17	19	2.91	35
		Posterior cingulate	30	10	−54	10	4.16	32,410
Temporal	Left	Superior temporal	22	−63	−44	8	2.75	44
Parietal	Left	Postcentral	5	−28	−41	65	2.94	208
		Postcentral	5	−18	−41	65	2.90	208
		Postcentral	7	−12	−47	61	2.33	208
	Right	Postcentral	7	12	−47	67	2.40	46
		Superior parietal lobule	7	26	−45	61	2.58	46
Sub-lobar	Left	Insula	13	−32	11	20	2.67	60
	Right	Thalamus		2	−18	21	2.44	19
Posterior cerebellum	Right	Declive		30	−69	−20	2.49	16

FWE *p* = 0.0167, corrected for multiple comparisons using cluster size = 12.

The ARND group had greater activation of the left middle frontal gyrus (BA 47), part of the ventrolateral prefrontal cortex, than ADHD subjects (Table 5). The only other regions where subjects with ARND showed greater activity than those with ADHD following the subtractive contrast included the right superior frontal (BA 10) and right middle temporal (BA 39) gyri (Figure 3, Table 5). There were no brain regions with greater functional activity in ARND subjects than the TD group following subtractive contrast.

Bilateral activation patterns were observed along both the ventral (BA 45 and 47) and dorsal (BA 3, 4, and 6) frontoparietal attention networks in TD subjects and to a lesser extent in ADHD subjects. The TD subject group had activations in areas along the TPJ, linking the two attention networks, centered at BA 22 and 40 and clearly extended to include BA 41, 42, 43, and 37 (Figure 2). Activations for the ARND group were much smaller, discrete points of activity, predominantly left-lateralized and confined to the frontal cortex at BA 10, 45, 46, and 47. There were no significant activations found within the parietal cortex and only one at the superior temporal gyrus (BA 38), representing a striking difference from the extensive activations observed in the TD group (Figures 2 and 3).

#### ARND/ADHD^+^

**Figure 4:**
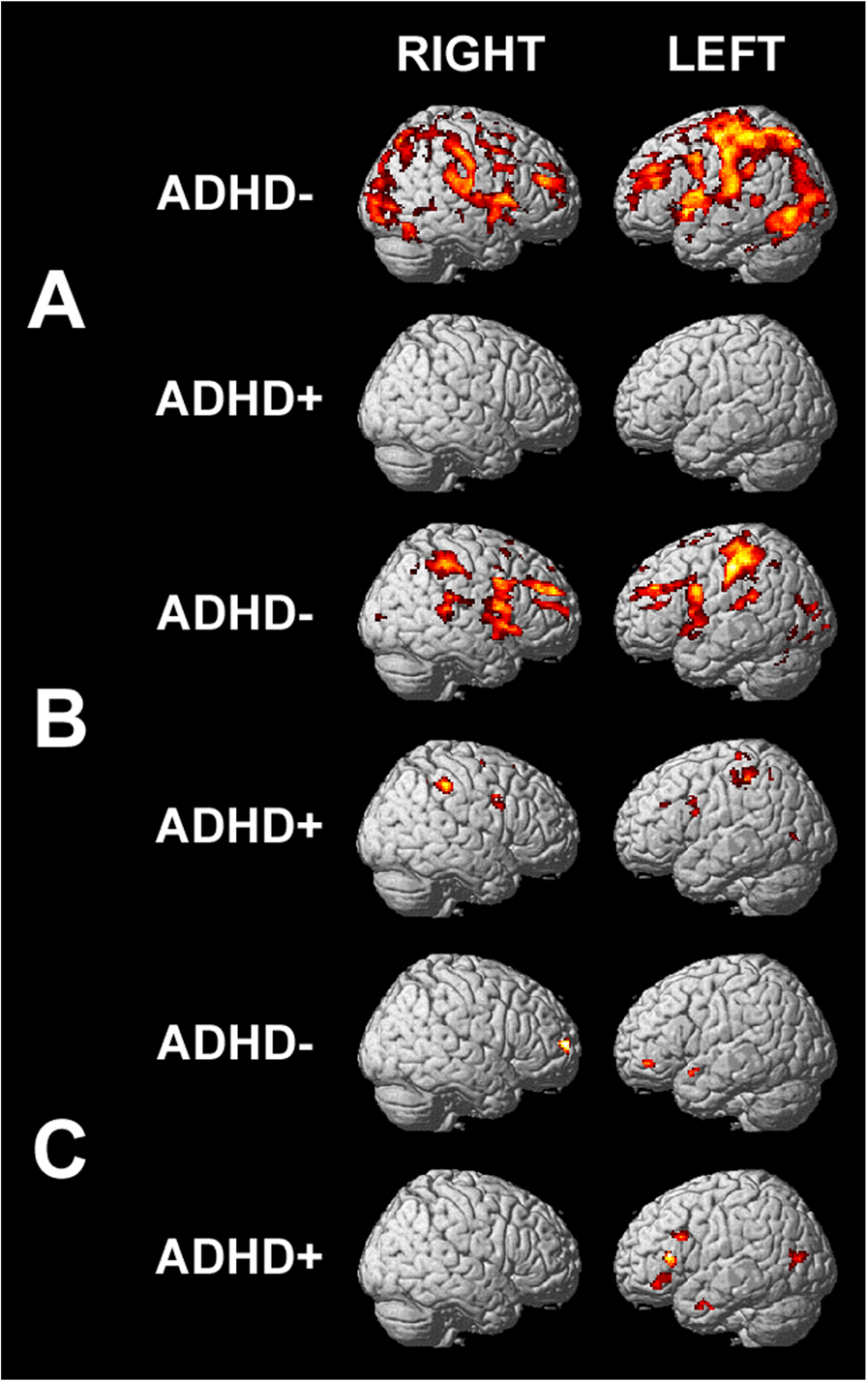
**fMRI activation maps for ARND subjects with and without diagnoses of ADHD. (A)** Disjunction contrast (FWE *p* = 0.001, corrected for multiple comparisons using cluster size = 10). **(B)** Conjunction contrast (FWE *p* = 0.001, corrected, cluster size = 10). **(C)** Conjunction minus disjunction contrast (FWE *p* = 0.05, corrected, cluster size = 22). R, right; L, left. The color of the activations corresponds to increased significance with increasingly hot colors (black-red-yellow-white); activations range from *T* = 1.9 (*p* = 0.05) for **(C)** and *T* = 3.9 (*p* = 0.001) for **(A)** and **(B)** to a maximum of *T* = 14.

Nine of the twenty-two children in the ARND group had a comorbid diagnosis of ADHD. Activation maps for ARND subjects with and without a comorbid diagnosis of ADHD for the conjunction, disjunction, and the subtractive (conjunction minus disjunction) contrasts are presented in Figure 4. Activation patterns for both disjunction and conjunction visual search were much larger for the ARND/ADHD^−^ subgroup than the ARND/ADHD^+^ subgroup. During disjunction search, the ARND/ADHD^+^ subgroup activated the right cuneus (BA 18; Figure 4A). This finding is in contrast to the ARND/ADHD^−^ subgroup which showed much greater cortical activations along the dorsal frontoparietal attention network, including bilateral frontal (BA 6, 8, 9, and 10), bilateral precentral (BA 4 and 6), left superior parietal (BA 7), bilateral superior temporal (BA 22), and occipital (BA 19) regions. For the conjunction search task, both subgroups activated task-relevant areas (BA 6, 9, 40, and 48; Figure 4B); however, ARND/ADHD^+^ activations were less intense. The subtractive analysis (Figure 4C) showed greater activations in areas of the frontal cortex (BA 9, 45, 46, and 47) and posterior middle temporal gyrus (BA 38) in ARND/ADHD^+^ subjects than ARND/ADHD^−^. Notably, there were no significant differences in subject characteristics or task performance (data for the ARND group as a whole are given in Tables 1 and 2, respectively) among the two subsets of the ARND group.

#### FSIQ

Because the FSIQ for the ARND group was lower than that of the TD group (Table 1), it was important to assess the contribution of FSIQ to group differences. It was also important to ascertain that the ARND subjects with the lowest FSIQ scores were not exerting undue influence on the result of the ARND group. Four subjects in the ARND group had FSIQ scores below 70, the threshold for diagnosis of cognitive impairment. In the visual search task of the conjunction array, all of these subjects had accuracy scores, response times, and response time variability less than 1.5 standard deviations from the ARND group mean. One ARND subject had an accuracy score greater than two standard deviations from the mean for the disjunction search. This same subject performed well on the more challenging conjunction task. Since all subjects performed well on the more challenging task, we felt that there was no reason to exclude any of the low-IQ ARND subjects based on their performance. To assess whether these subjects were outliers with respect to the fMRI data, the single-group analyses were repeated excluding these subjects. Activation maps for both tasks (data not shown) were essentially identical to those including all ARND subjects.

#### Age

Because age was well-matched among the groups (Table 1), a between-groups effect of age was not expected. However, the age range in this study corresponded to a broad developmental period during which brain development, especially in the frontal lobes, is ongoing [31] and could introduce the possibility of within-groups effects of age. Calculation of the *Z* score of conjunction search accuracy by grouping subjects according to age did not change significant relationships between the different groups. Conjunction: TD-ADHD *Z* = −1.966, *p* > 0.0167; TD-ARND *Z* = −5.792, *p* < 0.0167; ADHD-ARND *Z* = −2.910, *p* < 0.0167. Disjunction: TD-ADHD *Z* = −0.036, *p* > 0.0167; TD-ARND *Z* = −2.561, *p* < 0.0167; ADHD-ARND *Z* = −2.603, *p* < 0.0167. Although this resulted in a narrowing of all groups’ accuracy scores for disjunction search, the differences between them remained statistically significant. The activation maps for conjunction search and the subtractive analysis were unchanged by the inclusion of age as a covariate (data not shown). These results suggest that the range of subject ages did not obscure activations observed in fMRI analyses of the visual search task.

### DTI

**Figure 5:**
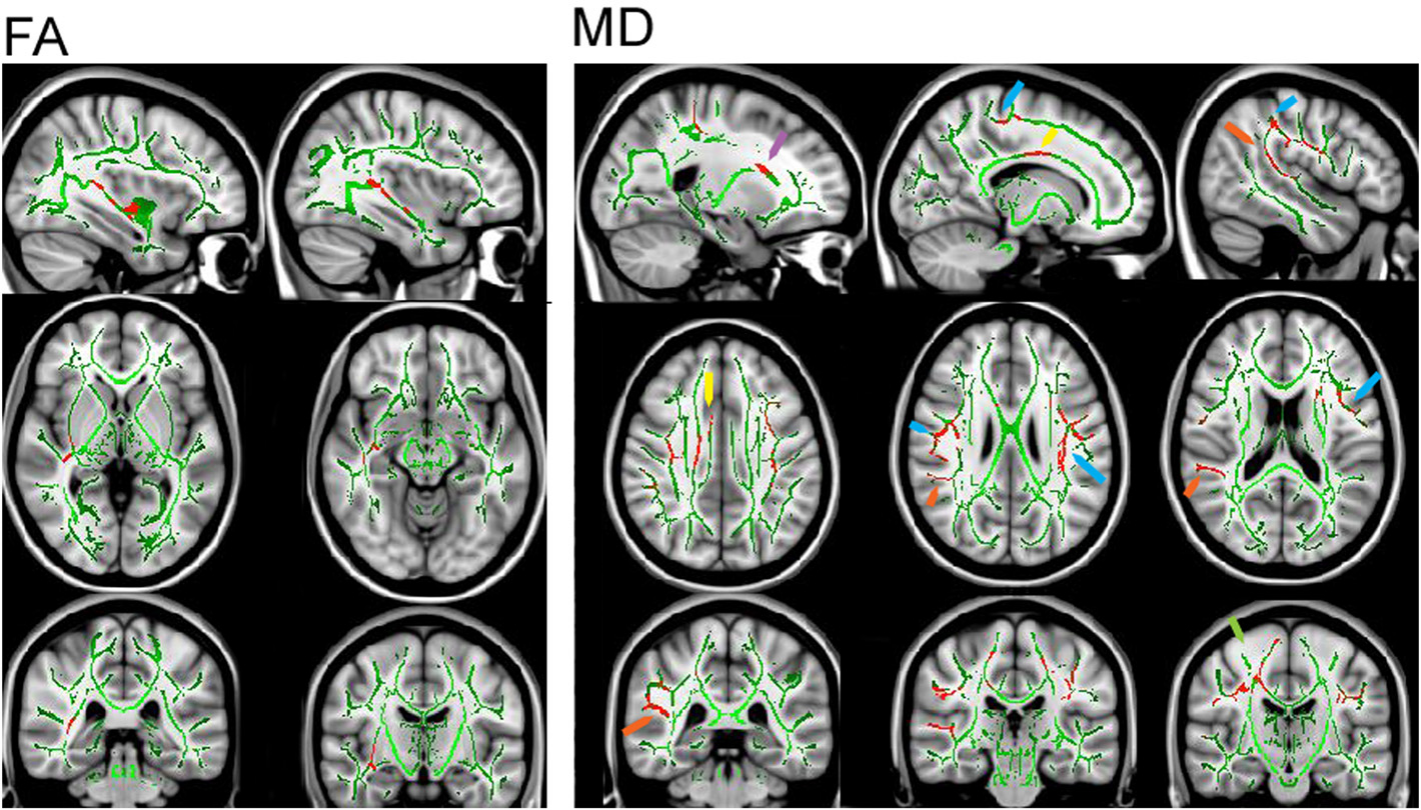
**TBSS analysis showing areas of significant differences in FA and MD between the ARND and TD groups.** The FA map (left) shows significance in the right ILF only. The MD maps (right) show significant differences in the ILF (orange arrow), SLF (blue arrow), IFO/UNC (purple arrow), CG (yellow arrow), and CC with projection to frontal regions (green arrow).

TBSS analysis showed areas of significantly greater FA (*p* < 0.05), the directional portion of diffusion, in the right ILF in TD compared to ARND subjects (Figure 5). No areas were found to have significantly higher FA in the ARND group compared to TD. MD, the average diffusion in all directions, values were found to be significantly greater for ARND than TD in several areas including the bilateral ILF; bilateral superior longitudinal fasciculus (SLF); corticospinal tract (CST); corpus callosum (CC); cingulate; external capsule; uncinate fasciculus (UF); inferior fronto-occipital fasciculus; superior corona radiata; arcuate fasciculus; and frontal, frontocentral, and central association fibers. No regions were found where MD was greater in the TD than ARND subjects (Figure 5). No significant differences were observed between ARND and ADHD or between TD and ADHD groups. Areas of significant differences between ARND and TD groups for both FA and MD overlapped within an area of the right ILF.

## Discussion

Deficits in attention and impulsive response style are known to be hallmark features of prenatal alcohol exposure and ARND [1,7,15]. While all subjects performed the fMRI tasks well, ARND subjects were less accurate than TD and ADHD subjects on both conjunction and disjunction searches (Table 2), highlighting the expected performance deficits characteristic of children diagnosed with ARND.

Although microstructural abnormalities may occur in many areas of the brain, the parietal lobe, posterior temporal areas, and the cerebellum seem particularly vulnerable to alcohol teratogenesis [10,32,33]. Notably, these sensitive areas play major roles in attentional processing and reveal connections to other areas of the cortex that are affected by prenatal alcohol exposure [17,33-37]. Directly in line with our hypothesis, subjects with ARND demonstrated significant activations in attention-related regions of the brain during the disjunction task compared to ADHD and TD controls. They appeared unable to utilize the same automatic mechanisms of attention that were demonstrated by the ADHD and TD subjects for this simple task. The conjunction search task resulted in significantly greater brain activations in ADHD and TD groups compared to ARND subjects. There was notably reduced activation in the ventral frontoparietal network and absence of differential TPJ activation in ARND compared to ADHD and TD subjects. This is consistent with interruption in the ventral frontoparietal network between the inferior frontal gyrus and the TPJ. The fMRI results are further supported by TBSS analysis demonstrating reduced FA and elevated MD in the ILF of ARND compared to TD subjects. Damage to the white matter of the ILF may compromise the ventral attention pathway. This would lead to problems in the simple bottom-up attention process required to detect targets in feature disjunction arrays, as seen in ARND subjects in the current study. Compromise of the ventral pathway may force subjects to use the dorsal pathway, which is associated with effortful top-down processing and required for conjunction search, for tasks that should be automatic, such as the feature disjunction task. This supports our hypothesis that the mechanisms of attention are different for ARND as compared to ADHD subjects.

### Attention network deficit in ARND

All three groups activated expected areas of the dorsal and ventral frontoparietal networks during both attention tasks. However, the similar levels of activation found in ARND children for both feature-based search tasks suggests that children with ARND reach their maximal level of effort at a lower task difficulty than either TD or ADHD children. The fMRI results may indicate that the more difficult conjunction search required more effort than the ARND subjects were able to provide. This is also reflected in their reduced accuracy scores (Table 2).

Clearly, the most striking result of these analyses is the extensive activity along both dorsal and ventral attention networks that is specific to conjunction search (i.e. following subtraction of disjunction from the conjunction array) in TD and ADHD groups compared to the ARND group (Figure 2C). As expected, TD subjects showed substantially greater activation within both dorsal and ventral attention networks, including the TPJ, during the more difficult conjunction task (Figure 2C). ADHD subjects also showed significantly enhanced dorsal attention pathway activity during the conjunction task compared to the disjunction task (Figure 2C). However, in contrast to both ADHD and TD subjects, the ARND group had significantly reduced activations in the subtractive contrast. This was primarily due to the extensive activations in the disjunction contrast found in the ARND group (Figure 2A). The subtractive contrast offers insight into attentional networks in the brain, as it isolates the only difference between tasks: the level of feature-related distraction (i.e., non-target items that share one feature with the target and therefore compete for attention). Attending to and selecting a target recruits both the dorsal and ventral frontoparietal attention networks, which are linked at the TPJ [23]. Firstly, the dorsal system would be required to search an array and secondly the ventral system would be utilized when unexpected items were encountered in the search process [18,38]. This suggests that the TPJ would be activated in typical controls to mediate between these two systems to allow goal-directed target detection during a feature conjunction task.

Activations common to TD and ARND subjects in the subtractive contrast were noted in the inferior frontal gyrus, which is responsible for connecting the dorsal and ventral frontoparietal networks and is involved in evaluating the salience of a stimulus and in triggering processing by the TPJ to determine its behavioral relevance [23]. The ventrolateral prefrontal cortex, which includes this region, is thought to play a role in comparison of stimuli and response selection [39] and in detection of unexpected stimuli at searched locations [23].

As mentioned, the dorsal parietal area belongs to the dorsal attentional network, which is associated with top-down processing. This region was activated during both visual search tasks in the ARND group. This extensive activation suggests over-deployment of focused attention to solve the relatively simple disjunction search. By contrast, this area was differentially activated in the TD group as a function of task; greater activation was observed during the conjunction search compared to the disjunction search, consistent with the expectation of differential cortical engagement as a function of attentional load. ADHD subjects demonstrated a similar pattern of activity in the subtractive contrast as TD subjects, although to a lesser extent.

The subtractive contrast revealed extensive parietal and temporal (including TPJ) activations for TD and ADHD groups compared to the ARND group. However, differential activation was noted in frontal components of the ventral pathway in the ARND group as a function of task. The greater frontal activity apparent in the subtractive contrast in ARND subjects indicates additional ventral pathway load. This is consistent with ARND subjects using the same attentional process for both tasks instead of increased utilization of the top-down attention mechanism as the task becomes more complex, as was evidenced by dramatically increased dorsal as well as ventral attention network activity in both TD and ADHD groups. The ARND group activated the superior temporal and inferior frontal gyri, both of which are integral to detection of unexpected or behaviorally relevant stimuli [23]. It is possible that subjects diagnosed with ARND were processing only one of the two feature cues (color or letter) during the target phase of the task. This would lead to activation of the bottom-up pathway by virtue of the presence of unexpected distractor stimuli that share that feature with the feature conjunction target.

The superior temporal gyrus has been shown to be involved with motor response generation, working memory, decision making, and the disengagement and orientation of attention. These are all components of target search and detection in combined feature arrays [27]. Activation within the superior temporal gyrus by all groups suggests that similar processing mechanisms were used; however, activation of the superior temporal gyrus in ARND subjects was only found in a more anterior and ventral position (BA 38). This unexpected activation location suggests that cortical processing along the ventral frontoparietal network is interrupted somewhere between the inferior frontal gyrus and the TPJ.

Compared to the ARND group, the TD group showed increased activations throughout the ventral frontoparietal network in the subtractive contrast (Figure 2C). These activations included sub-gyral white matter activations at BA 21 and gray matter activations at BA 22, 40, 2, and 3. The ADHD group also activated BA 40, 2, and 3, which are located in the direct vicinity of the TPJ. The absence of differential TPJ activation in the ARND group compared to the other two groups in the subtractive contrast is striking, especially considering its importance in regulating attentional pathways. The failure to differentially activate this region in subjects diagnosed with ARND may therefore reflect a neurological limit to the effective utilization of both attentional strategies.

The lack of functional activation in the vicinity of the TPJ may be directly related to deficits in white matter integrity along the right ILF determined by DTI (Figure 5). The ILF is a long association fiber bundle that connects the occipital lobe to the inferior temporal region and interacts with the UF, allowing extended connection to the frontal lobe.

TBSS analysis revealed significantly reduced FA along the right ILF in individuals diagnosed with ARND compared to TD subjects (Figure 5). No other significant FA differences existed elsewhere in the brain in ARND compared to TD, and no differences were observed between ARND and ADHD. Areas of higher MD were also observed in ARND subjects compared to TD subjects. Regions of elevated MD not only overlapped with that of low FA in the right ILF but also extended into the arcuate component of the SLF that links superior temporal regions with the inferiolateral frontal cortex (Figure 5). Both FA and MD are sensitive to microstructural changes to white matter integrity and organization. FA is the directional component of diffusion, while MD is the average diffusion in all directions. Typically, MD is very similar for gray and white matter and is higher for CSF. Interpretation of DTI is complex, and individual results of reduced FA or elevated MD are not necessarily conclusive [40]. Areas of overlap between these measures provide more confidence in these results, especially given the limitations in acquisition parameters (i.e., non-isotropic voxels) in such a difficult group of subjects to image. Reduced FA and elevated MD reflect axonal damage or conditions that adversely affect axonal development [32,41]. Taken together, the DTI results are consistent with impairment in functional connectivity between posterior and anterior components of the network controlling attention. This impairment in subjects diagnosed with ARND seems to be co-localized with regions of the TPJ that failed to activate appropriately to support increased attentional strategies in the conjunction search tasks.

Sowell et al. also observed reduced FA and high MD in FASD/ARND subjects along the right ILF in addition to widespread microstructural differences between FASD and typical controls [31,42]. Serious deficits have been reported in ARND subjects in ILF integrity, which is the white matter tract responsible for connecting functional components of the ventral frontoparietal attention system including the corpus callosum, cingulate, and right temporal areas [32,41-45]. It is also well-established that white matter is sensitive to the teratogenic effects of alcohol and may be related to changes in myelination, axonal loss, axonal damage, or white matter disorganization during development [31,41,42].

To our knowledge, no studies of individuals diagnosed with ADHD, including the present study, have shown reduced FA or increased MD in the right ILF as was observed in individuals diagnosed with ARND. Poor integrity of white matter along the right ILF linked to the ventral frontoparietal attention network may be in part responsible for attention deficits characteristic of children diagnosed with ARND. This suggests a different mechanism of attentional disruption in subjects diagnosed with ARND linked to problems with white matter integrity in this region, which is absent in ADHD populations.

While the only difference in FA observed in the present study was in the right ILF in the ARND group compared to TD subjects, there were several other regions, in addition to the ILF and CC, that showed elevated MD in subjects diagnosed with ARND compared to TD. Although others did not find significant MD increases in the UF, a trend toward elevated MD in the right hemisphere was observed in individuals diagnosed with FASD [43], and Fryer et al. noted lower FA in the UF of FASD subjects with heavy prenatal exposure to alcohol [44]. Compromised integrity of the UF, particularly in the right hemisphere of ARND subjects, may add to the problems with attention noted in ARND subjects.

### ARND subjects with diagnoses of ADHD

ADHD symptomatology is common among children with FASD; however, symptoms may differ qualitatively between subjects with and without fetal alcohol exposure. Children with FASD perform worse on tests assessing their ability to encode new information and to shift attention from one set of rules to another, whereas children with ADHD tend to perform worse on tests assessing their ability to focus and sustain their attention and their ability to retrieve learned information [6,46].

In the past, there has been confusion about whether attention problems in FASD are part of FASD or whether this represents a different diagnosis of ADHD. Previous terminology has been problematic and may account for the large range of FASD/ADHD comorbidity published in the literature. However, as pointed out by Lane et al., while subjects with prenatal exposure to alcohol consistently demonstrate behavioral symptoms of inattention, they do not necessarily demonstrate deficits on experimental or clinical measures of attentional functioning [47]. Our proportion of ARND/ADHD comorbidity is consistent with that found in the literature [13].

Nine of the 22 children in the ARND group had a comorbid diagnosis of ADHD. The activation patterns for both disjunction and conjunction visual search were much stronger for the ARND/ADHD^−^ subgroup than the ARND/ADHD^+^ subgroup. The intensity of activations for the disjunction search in ARND/ADHD^−^ subjects strongly contrasts that found for the ARND/ADHD^+^ subgroup and for the clinical ADHD group (Figures 2A and 4A). Activation patterns in ADHD subjects that were found to be over and above those of the TD group for the conjunction search did not correspond to the few areas of functional activity seen in the ARND/ADHD^+^ subgroup. This finding suggests that the ARND/ADHD^+^ subgroup activations may not be attributed to the same attentional deficits as found in the ADHD clinical population.

### Study limitations

A few limitations of the study are important to note. The task was designed to be simple so that all subject groups would be able to adequately perform the tasks. Caution should be exercised in reaching any conclusions that there are only small differences among these groups in attention from our performance data.

Children diagnosed with ARND included in our study were evaluated carefully and confirmed to have a history of prenatal alcohol exposure; however, it is also possible that illicit drugs were also used during pregnancy. The use of illicit drugs was not used as an exclusion criterion for our study because of the high co-occurrence with prenatal alcohol exposure. It was undesirable to restrict participation in the study for this reason, as it may have resulted in sample bias. Other postnatal environmental factors may have affected the ARND subjects’ neurodevelopment. Lower SES may have resulted in poorer environmental enrichment and nutritional status, both factors that can affect neurodevelopment.

While there are several other TBSS analyses now in the literature, much of the work in FASD has been general region of interest analysis of DTI. TBSS is a voxel-based method that examines the center of white matter tracts, whereas region of interest analysis examines the entire tract in the region of interest. There may be differences in DTI measures at the edges of these pathways compared to the centers. However, the use of a voxel-based method, such as TBSS, allows for characterization of the white matter across the entire brain and direct comparison on a voxel-by-voxel basis between groups. It should also be noted here that the TBSS analysis parameters used were optimized to acquire reliable data in affected populations of children who were unable to remain still for prolonged periods of time. Therefore, in order to acquire the data of the whole brain quickly, larger than optimal slice thickness was used. As a result, partial voluming effects cannot be ruled out. However, the information obtained from the subjects was of extremely high quality with minimal motion allowing for accurate mapping. Additionally, since TBSS analysis concentrates on the center of the white matter tracts, we believe any partial voluming effects to be minimal. Additionally, it should be noted that TBSS analysis as a probabilistic method is better at avoiding problems involving tract crossings and acute angles compared to deterministic methods. It will be important to corroborate these TBSS results using thinner slice thickness (2 mm; isotropic voxels) concentrated on the region of interest we now believe to be crucial in attentional problems related to ARND (i.e., ILF and TPJ) with a recommended minimum group size of 30 subjects.

It should also be noted that we do not believe any of the previously performed DTI studies involving ADHD subjects included screening for FASD. Several studies cited in this paper have documented the involvement of subjects with comorbid diagnoses. We are confident that our screening procedures have allowed for accurate representation of the groups ensuring accurate ADHD diagnosis.

## Conclusions

The neural mechanisms behind attentional deficits commonly reported for children with ARND are critical for understanding FASD. Convergence of the functional activation patterns during an attention task employing conjunction and disjunction searches and reduced white matter tract integrity suggests a deficit in the processing of attentional information in ARND subjects. In TD subjects, and to a lesser extent in ADHD subjects, functional activations related to target detection in conjunction arrays (i.e., the subtractive contrast) engage important cortical areas of the brain belonging to both dorsal and ventral frontoparietal attentional networks. In contrast, ARND subjects reveal activation patterns only within anterior portions of the brain, specifically along the anterior portion of the ventral frontoparietal network. The reduced activation in ARND subjects during the conjunction task (subtractive contrast) is related to the extensive functional activations observed in this group during the simple disjunction task. Children diagnosed with ARND appear unable to effectively use the very efficient automatic perceptual pop-out mechanism employed by the TD and ADHD groups during presentation of the disjunction array.

The notably reduced functional activation in the ventral frontoparietal network in ARND subjects and absence of differential TPJ activation in ARND compared to ADHD and TD subjects is consistent with interruption in the ventral frontoparietal network between the inferior frontal gyrus and the TPJ. This is further supported by the TBSS analysis, which demonstrated poor integrity of the ILF in ARND compared to TD subjects and may result in an interruption in attentional processing along the ventral network.

The current study provides substantial evidence that teratogenic exposure to alcohol can cause neurophysiological developmental problems responsible for attentional deficits in children with ARND that appear to arise from different attentional mechanisms than in subjects diagnosed with ADHD.

## Abbreviations

AC-PC: anterior commissure-posterior commissure
ADHD: attention-deficit/hyperactivity disorder
ANCOVA: analysis of covariance
ANOVA: analysis of variance
ARND: alcohol-related neurodevelopmental disorder
ASI: Adolescent Symptom Inventory
BA: Brodmann area
BRIEF: Behavior Rating Inventory of Executive Function
CC: corpus callosum
CNS: central nervous system
CPT: Continuous Performance Test
CSI: Child Symptom Inventory
CST: corticospinal tract
DSM: Diagnostic and Statistical Manual
DTI: diffusion tensor imaging
EPI: echo-planar imaging
FA: fractional anisotropy
FAS: fetal alcohol syndrome
FASD: fetal alcohol spectrum disorder
fMRI: function magnetic resonance imaging
FOV: field of view
FSIQ: full-scale intelligence quotient
FWE: family-wise error
HSD: honestly significant difference
HRF: hemodynamic response function
ILF: inferior longitudinal fasciculus
IQ: intelligence quotient
ISI: interstimulus interval
MD: mean diffusivity
MNI: Montreal Neurological Institute
MPRAGE: magnetization prepared rapid gradient echo
MRI: magnetic resonance imaging
ODD: oppositional defiant disorder
OM: errors of omission
PPC: posterior parietal cortex
ROI: region of interest
SE: spin-echo
SES: socioeconomic status
SLF: superior longitudinal fasciculus
SPSS: Statistical Package for Social Sciences
TBSS: tract-based spatial statistics
TD: typically developing
TE: echo time
TI: inversion time
TPJ: temporoparietal junction
TR: repetition time
UF: uncinate fasciculus
WISC: Wechsler Intelligence Scale for Children
WMI: Working Memory Index
